# Evaluation of Publication of COVID-19–Related Articles Initially Presented as Preprints

**DOI:** 10.1001/jamanetworkopen.2022.45745

**Published:** 2022-12-08

**Authors:** Carl Llor, Ana Moragas, Manfred Maier

**Affiliations:** 1 Research Unit for General Practice, Department of Public Health, University of Southern Denmark, Odense, Denmark; 2 Primary Care Research Institute Jordi Gol, Barcelona, Spain; 3 Centro de Investigación Biomédica en Red de Enfermedades Infecciosas, Instituto de Salud Carlos III, Barcelona, Spain; 4 Jaume I Health Centre, Institut Català de la Salut, University Rovira i Virgili, Catalonia, Spain; 5 Department of General Practice and Family Medicine, Centre for Public Health, Medical University of Vienna, Vienna, Austria

## Abstract

This cross-sectional study evaluates subsequent journal publication of COVID-19–related articles initially posted as medRxiv preprints in 2020.

## Introduction

Since the launch of the medRxiv preprint server in 2019, the dissemination of research as preprints has grown rapidly, largely facilitated by the COVID-19 pandemic.^1^ Notwithstanding, this unprecedented increase in preprints has been subject to criticism, mainly because of reliability concerns owing to their lack of peer review. In 2020, Abdill et al^2^ reported that 62.6% of bioRxiv preprints were later published in scientific journals, considering a time frame of at least 1 year. However, other studies^3,4^ have highlighted the low percentage of medRxiv preprints subsequently published in journals, with publication rates of 14.0% after 0 to 12 months^3^ and 10.6% after 6 to 19 months.^4^ In an analysis of COVID-19–related preprints posted on 3 servers, Añazco et al^5^ observed that 5.7% were published in a journal 3 to 8 months after their preprint posting. To our knowledge, no recent studies have analyzed whether journal publication rates of medRxiv preprints have changed. Therefore, we conducted this study to evaluate the subsequent journal publication of COVID-19–related preprint articles posted on medRxiv in 2020.

## Methods

This cross-sectional study did not require institutional review board approval or informed consent because it used publicly available data, in accordance with 45 CFR §46. The study followed the STROBE reporting guideline.

In March 2022, we searched preprints on medRxiv and included all papers on COVID-19 in the infectious diseases subject area that were posted between January 1 and December 31, 2020. We repeated this search in October 2022. Two of us (C.L. and A.M.) completed and verified both searches.

We checked whether a preprint was already published in a peer-reviewed journal by measuring the proportion of medRxiv preprints flagged as published. We recorded the journal names and obtained the journal rankings by measuring their quartile according to the updated 2021 Journal Citation Reports,^6^ in which quartiles 1 and 4 indicate the top 25% and bottom 25% of journals in a particular category, respectively.

Results are presented as counts and percentages. Descriptive statistical analyses were conducted using Excel software, version 16.0 (Microsoft Corp).

## Results

In this study, we identified 3343 COVID-19–related preprints posted on medRxiv in 2020. Our March 2022 search indicated that 1712 of those preprints (51.2%) were subsequently published in the peer-reviewed literature; this number increased to 1742 (52.1%) when we repeated the search in October 2022. Not considering January 2020, in which only 1 article on COVID-19 was posted, the rate of subsequent publication in a scientific journal ranged from 43.5% (94 of 216 preprints; observed in March 2020) to 60.6% (177 of 292 preprints posted in August 2020). The Table shows the top 25 of 579 peer-reviewed journals in which these preprints were published; 827 preprints (47.5%) were subsequently published in quartile 1 journals (Figure).

**Table.** Top 25 Peer-Reviewed Journals in Which 2020 COVID-19–Related medRxiv Preprints Were Subsequently Published, by Number of Papers

Rank	Journal	No. of 2020 preprints published	2021 journal impact factor	Quartile (subject area)
1	*PLoS ONE*	152	3.752	Q2 (multidisciplinary sciences)
2	*Scientific Reports*	52	4.996	Q2 (multidisciplinary sciences)
3	*BMJ Open*	44	3.006	Q2 (medicine, general and internal)
4	*Nature Communications*	44	17.694	Q1 (multidisciplinary sciences)
5	*Clinical Infectious Diseases*	39	20.999	Q1 (immunology; infectious diseases; microbiology)
6	*Journal of Medical Virology*	37	20.693	Q1 (virology)
7	*Journal of Clinical Microbiology*	34	11.677	Q1 (microbiology)
8	*International Journal of Infectious Diseases*	32	12.074	Q1 (infectious diseases)
9	*BMC Infectious Diseases*	24	3.667	Q3 (infectious diseases)
10	*Journal of Clinical Virology*	23	14.481	Q1 (virology)
11	*Clinical Microbiology and Infection*	21	13.310	Q1 (microbiology; infectious diseases)
12	*Frontiers in Medicine*	21	5.058	Q2 (medicine, general and internal)
13	*Emerging Infectious Diseases*	18	16.126	Q1 (infectious diseases; immunology)
14	*Journal of Infectious Diseases*	18	7.759	Q1 (infectious diseases; microbiology; immunology)
15	*eLife*	15	8.713	Q1 (biology)
16	*Frontiers in Immunology*	14	8.786	Q1 (immunology)
17	*Journal of Infection*	14	38.637	Q1 (infectious diseases)
18	*Science of the Total Environment*	14	10.753	Q1 (environmental sciences)
19	*Journal of Virological Methods*	13	2.623	Q3 (biochemical research methods) and Q4 (virology; biotechnology and applied microbiology)
20	*Open Forum Infectious Diseases*	12	4.423	Q2 (infectious diseases; microbiology) and Q3 (immunology)
21	*Annals of Translational Medicine*	11	3.616	Q3 (medicine, research and experimental; oncology)
22	*eClinicalMedicine*	11	17.033	Q1 (medicine, general and internal)
23	*International Journal of Environmental Research and Public Health*	11	4.614	Q1 (public, environmental, and occupational health) and Q2 (environmental sciences)
24	*Proceedings of the National Academy of Sciences USA*	11	12.779	Q1 (multidisciplinary sciences)
25	*Viruses*	11	5.818	Q2 (virology)

Abbreviation: Q, quartile.

**Figure.**
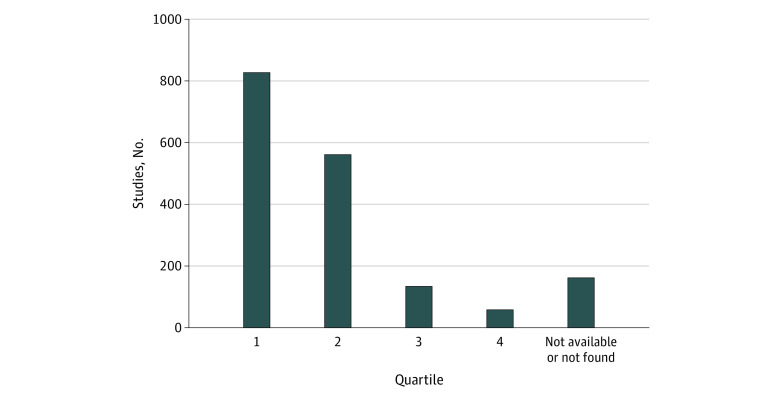
Quartile Rankings of Peer-Reviewed Journals in Which 2020 COVID-19–Related medRxiv Preprints Were Subsequently Published

## Discussion

Researchers are able to communicate their findings immediately by posting papers on preprint servers, which has become even more important during the COVID-19 pandemic. In this cross-sectional study, we observed that slightly more than half of the preprints related to COVID-19 posted on medRxiv in 2020 were later published in peer-reviewed journals as of October 2022. This publication rate is only slightly greater than that observed 7 months earlier in March 2022, which suggests that a substantial change in the proportion of papers subsequently published in peer-reviewed journals is not expected in the future. Another notable finding of this study is the high quality of the journals in which these articles were subsequently published, as nearly half of the preprints were published in quartile 1 journals.

This study has the limitation of having analyzed only preprints related to COVID-19 posted on a single preprint server. The publication rate of preprints on other topics may be different. Future studies aimed at evaluating publication rates in other areas of medical science are needed.

## Notes

Supplement.
Data Sharing Statement
