# Incomplete Sentence in Discussion Section

**DOI:** 10.1001/jamanetworkopen.2023.0341

**Published:** 2023-02-08

In the Research Letter titled “Evaluation of Publication of COVID-19–Related Articles Initially Presented as Preprints,”^1^ published December 8, 2022, a sentence in the Discussion concerning the study’s limitations was incomplete. In the second paragraph of the Discussion section, the first sentence should have read as follows: “This study has the limitations of having analyzed only preprints related to COVID-19 posted on a single preprint server and relying only on the medRxiv indication of a related published article, which may have resulted in undercounting.” This article has been corrected.^1^
