# Supplementary material for: Evaluation of Publication of COVID-19–Related Articles Initially Presented as Preprints
Source: JAMA Netw Open. 2022 Dec 8;5(12):e2245745. doi: 10.1001/jamanetworkopen.2022.45745 (PMC9856219; doi:10.1001/jamanetworkopen.2022.45745)
Supplement: Supplement. — Data Sharing Statement [file jamanetwopen-e2245745-s001.pdf]

Data Sharing Statement

Llor. Evaluation of Publication of COVID-19-Related Articles Initially Presented as Preprints. *JAMA Netw Open*. Published December 08, 2022. doi:10.1001/jamanetworkopen.2022.45745

Data
Data available: No
